# Supplementary material for: A Whole Genome Sequencing-Based Epidemiological Investigation of a Pregnancy-Related Invasive Listeriosis Case in Central Italy
Source: Pathogens. 2022 Jun 8;11(6):667. doi: 10.3390/pathogens11060667 (PMC9228178; doi:10.3390/pathogens11060667)
Supplement: Supplementary file 1 [file pathogens-11-00667-s001.zip › Supplementary Figure S1.pdf]

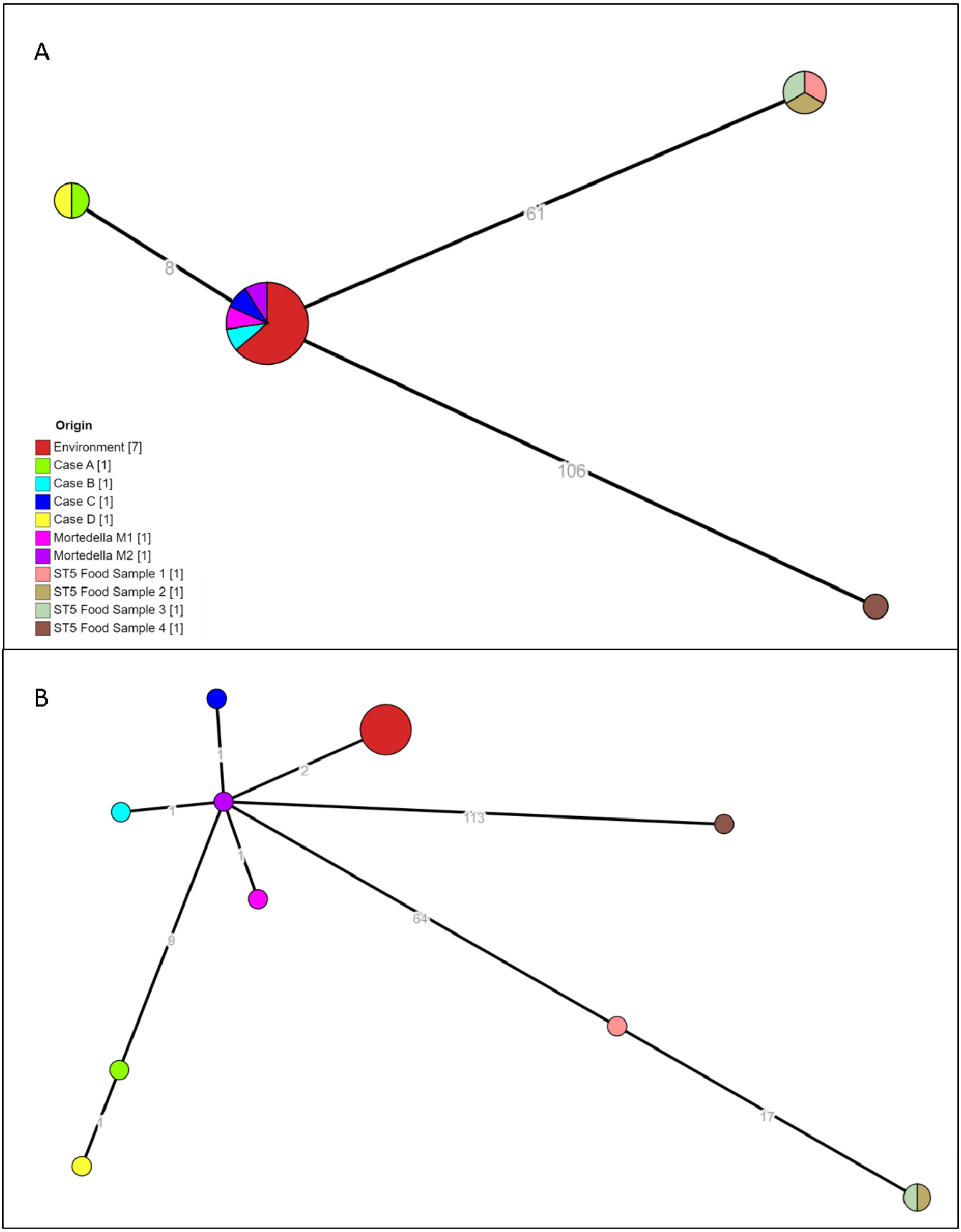

Figure S1. Core genome MLST characterizations of isolates from the main investigation and other ST5 food samples, assessed with the two softwares cgMLSTFinder (**A**) and chewBBACA (**B**).
